# How Antiparasitic Drugs Work—And Sometimes Stop Working!

**DOI:** 10.1371/journal.ppat.1005430

**Published:** 2016-05-05

**Authors:** David Horn

**Affiliations:** Division of Biological Chemistry and Drug Discovery, School of Life Sciences, University of Dundee, Dundee, United Kingdom; University of Notre Dame, UNITED STATES

As a molecular biologist, you start by learning the basics of the DNA code. Reading and translating the code come first, and then you can move on to doing your own careful editing using cut and paste and copy and paste. Once you’ve acquired these skills, you can manipulate genes, genomes, and proteins to examine how cells “work” and to adjust the way they work. The power, versatility, and affordability of these techniques have improved in leaps and bounds in recent years, in parallel with improvements in information technologies. For example, DNA sequencing, or the ability to “read” an entire genome, is now accessible to any reasonably well-resourced research team.

The techniques are important, but it’s really what you do with them that can capture the imagination. The discovery of key disease-associated genes, often many years after the consequences of their action, or inaction, are known, has been a source of inspiration to me personally. In particular, I recall reading about identification of the cystic fibrosis gene in 1989, when starting my PhD, and hearing about identification of the leptin gene, linked to obesity, as a post-doc in 1995. Then, I saw what impact gene discovery could have on my own molecular parasitology community: the malaria parasite *var* genes in 1995, the African trypanosome human serum resistance-associated gene in 1998, and the malaria parasite chloroquine resistance transporter in 2000 are just a few examples. It remains challenging, though, to identify genes involved in a particular process of interest. Discovery of a new and important gene is also only the first step in research aimed at fully understanding function and potential as a therapeutic target or a vaccination candidate, for example. The outstanding challenges, however, are exactly what continue to drive technological innovation in the life sciences.

So what’s the situation for the unicellular trypanosomatid parasites we work on in Dundee? These highly motile cells, about 1/50th of a millimetre long, are spread between mammals by blood-sucking insects. They have a devastating impact on the world’s poor, causing the “neglected tropical diseases” African trypanosomiasis, Chagas disease, and the leishmaniases. The consequences of this range of human and animal diseases are hundreds of thousands of deaths each year and around a million cases a year of the disfiguring lesions associated with cutaneous leishmaniasis. Trypanosomes also severely curtail the import of susceptible horses, cattle, sheep, and goats across Africa.

We’ve focussed on African trypanosomes in particular and see that antiparasitic adaptations in the host and antiparasitic drugs are constantly threatened by obsolescence in the “arms race” between us and the parasites. In the hope of helping to gain the upper hand in this race, we adapted a genetic screening approach that allows us to survey every parasite gene for roles in drug action and drug resistance. This revealed >50 new genes linked to drugs in clinical use. The drugs nifurtimox and eflornithine, for example, are widely used as a combination therapy. We confirmed that nifurtimox is converted to a more potent drug by a parasite enzyme and, as also reported independently by other groups, found that eflornithine is taken up by a parasite amino acid transporter.

A much older drug, suramin, was found to hitch-a-ride into the cell on a parasite surface protein. This was a particularly satisfying discovery because Paul Ehrlich, “the father of chemotherapy,” who developed both the “magic bullet” hypothesis and the precursors to suramin over 100 years ago, stated, “When once we are acquainted with the majority of the chemioreceptors of a particular kind of parasite…we shall have far-reaching possibilities of simultaneous attack by various agencies” (Ehrlich, 1913). So, we found one of Ehrlich’s “chemioreceptors,” and this does indeed present new opportunities for therapeutic development.

Another finding related to two more old drugs for which cross-resistance had been reported over 60 years ago. This melarsoprol and pentamidine cross-resistance was linked to a defect in an aquaglyceroporin, another parasite membrane transporter. Resistance to melarsoprol, a rather toxic but otherwise effective arsenic-containing drug, had been observed in up to 50% of patients in some areas. Teams that collected and stored resistant parasites from across Africa were able to rapidly identify defects in this transporter and to trace them back to the mid-1970s. Thus, the genetic changes that allowed trypanosomes to resist this particular therapy are now known, the impact of these changes can be investigated in detail, and the spread and distribution of resistant parasites can be effectively monitored.

It’s quite remarkable how biological processes frequently become experimentally accessible in unanticipated ways, probably because we still have so much to learn about many of them. This has also been my personal experience. In the case of our own findings, it was our work on DNA repair that allowed us to develop the genetic screening approach described above. In addition, our initial motivation for developing this approach was a desire to tackle a completely different gene expression question; fortunately, we were able to initiate collaborations with colleagues who were already established experts in drug resistance.

What is clear is that knowing how drugs work and how they stop working helps improve the prospects of devising more effective and durable therapies and also improves prospects for monitoring and tackling resistance when it arises. As primarily “basic researchers,” working with partners in the Dundee Drug Discovery Unit, we will continue our efforts to discover and dissect druggable biology in the trypanosomatids. Indeed, tools and technologies are now in place to probe these chemical–biology interactions at higher throughput than ever before.

Since our work continuously relies upon the work and discoveries of many colleagues—too many to mention, I’m afraid—I end by reiterating what’s probably rather obvious: impact against these and other human and animal pathogens requires a community of researchers, critical mass, open interaction, collaboration, and access to powerful technology, at both basic and translational levels. What is also clear is that experiments with pathogens will continue to yield insights of far broader relevance to the life sciences and beyond.

**Image 1:**
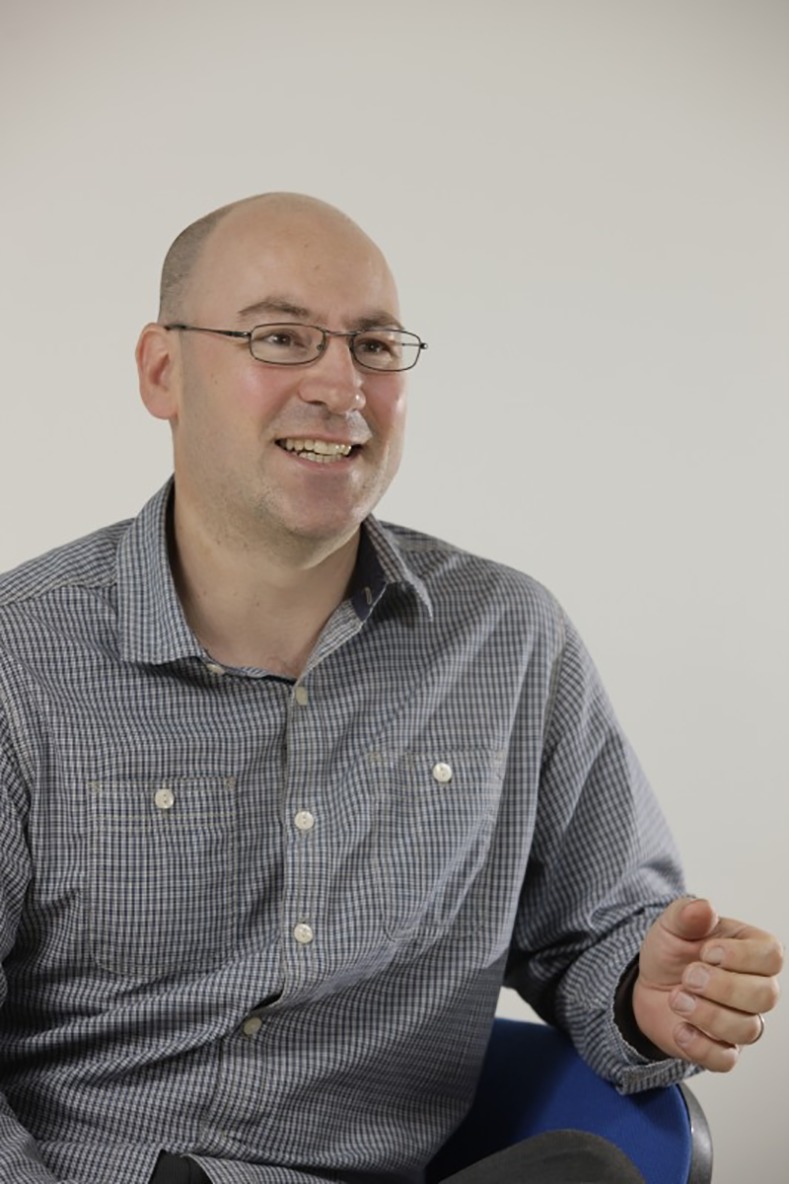
David Horn.

